# Novel prognostic biomarkers, METTL14 and YTHDF2, associated with RNA methylation in Ewing’s sarcoma

**DOI:** 10.1038/s41598-022-06744-0

**Published:** 2022-04-29

**Authors:** Jie Jiang, Qie Fan, Haishun Qu, Chong Liu, Tuo Liang, Liyi Chen, Shengsheng Huang, Xuhua Sun, Jiarui Chen, Tianyou Chen, Hao Li, Yuanlin Yao, Xinli Zhan

**Affiliations:** 1 The First Clinical Affiliated Hospital of Guangxi Medical University, Nanning, 530021 People’s Republic of China; 2 Department of Traditional Chinese Medicine, The People’s Hospital of Guangxi Zhuang Autonmous Region, Nanning, 530000 People’s Republic of China

**Keywords:** Cancer, Chemical biology, Immunology, Biomarkers, Oncology

## Abstract

Ewing’s sarcoma has a poor prognosis and high metastasis rate; thus, it is critical to explore prognostic biomarkers of m6A-related genes. Two datasets were downloaded from the Gene Expression Omnibus database, m6A-related genes were extracted, and prognostic models were constructed using the least absolute shrinkage and selection operator and multivariate COX regression analyses. Immune cell composition and drug sensitivity analyses were performed, and our analysis was validated using laboratory methods of immunohistochemical specific staining and qRT-PCR. Ewing’s sarcoma prognostic model demonstrated that the survival rate of cases in the high-risk group was much lower than that of the low-risk group. Naïve B cells, macrophages M0, macrophages M1, and resting mast cells are closely associated with Ewing’s sarcoma. METTL14 and YTHDF2 are strongly associated with multiple drug sensitivity. Immunohistochemical specific staining revealed higher expression of both METTL14 and YTHDF2 in Ewing’s sarcoma than in the paraneoplastic tissues. The results of qRT-PCR showed that METTL14 expression was significantly higher in both ES cell lines than in the control cell line. The prognostic model constructed using m6A-related genes METTL14 and TYHDF2, can be a potential prognostic biomarker for Ewing’s sarcoma, with the survival rate of cases in the high-risk group being much lower than that of the low-risk group.

## Introduction

Ewing’s sarcoma (ES) represent a newly emerging subgroup of small round cell sarcomas. They share several morphological, immunohistochemical, molecular, and clinical features and have recently become the second most common type of skeletal tumor^1^. This disease originates from mesenchymal stem cells, and it is the second most common sarcoma of the skeleton in young individuals and children. Further, this is also a highly aggressive type of mesenchymal tumor showing a very high metastatic rate^2–4^. The epigenetic tumor mechanisms have implications in mesenchymal tumors, chondroblastoma, epithelial sarcoma, and ES, with a higher prevalence of these diseases in young individuals^5^. Thus, possible disease prognostic biomarkers should be determined due to the high metastasis rate and poor prognosis.

The modifications in RNA methylation account for more than 60% of all RNA modifications, with those in N-6 methylation being the most prevalent among all higher biological lncRNAs and mRNAs. Previous studies have demonstrated that the complexity of cancer development can be determined by m6A methylation modifications by regulating cancer-related biological functions. The m6A modifications of the noncoding RNAs modulates the stability, cleavage, transport, and degradation of noncoding RNAs; and also monitors cancer cell proliferation, metastasis, homeostasis, and stem cell differentiation by altering the biological functions in cancer^6–8^. RNA m6A modifications regulate RNA splicing, allosterism, stability, and protein translation. The m6A is primarily catalyzed by RNA transferases METTL3, METTL14, and METTL16 (writers), cleared by demethylases FTO and ALKBH5 (scavengers), and interacts with m6A binding proteins such as YTHDF1 and IGF2BP1 (readers). RNA demethylases, methylesterases, and m6A binding proteins are also upregulated in various human cancer tissues. This increased the expression of cancer cell proliferation, survival, and metastatic tumor proteins^9^.

Immune cell infiltration indicates the modification of the immune cell microenvironment, which possesses the tumorigenesis site. Most tumor cells express antigens mediating recognition by host CD8 + T cells. Based on the molecular features and cells of the tumor microenvironment, tumor escape is classified into two main types. The first type, including the infiltrating T cells, is characterized by most inflammatory chemokines and innate immune activation of type I interferons. In contrast, the second is phenotyped as lacking this T cell inflammatory phenotype, possibly through rejection by the immune system to resist immune attack^10^. Moreover, in recent years, an increasing number of studies have shown that tumor immune cell infiltration plays a critical role in cancer^11–15^.

In the present study, we analyzed gene expression and survival data downloaded from the Gene Expression Omnibus (GEO) data using a combination of bioinformatics and laboratory validation to determine the role of m6A-related genes in ES. Furthermore, we aimed to provide new evidence by constructing prognostic models and analyzing immune cell composition and drug sensitivity to assist in clinical diagnosis and treatment.

## Materials and methods

### Data download and preliminary processing

The gene expression and clinical information data used for the present study were downloaded from the GEO (https://www.ncbi.nlm.nih.gov/gds/) database. Our selection criteria for datasets: 1. unique datasets with available clinical data and 2. all solid tumor specimens derived from Ewing's sarcoma. We downloaded two different datasets to make the analysis more logical and rigorous. We used GSE17674 (https://www.ncbi.nlm.nih.gov/geo/query/acc.cgi?acc = GSE17674)^16^, and GSE63157 (https://www.ncbi.nlm.nih.gov/geo/query/acc.cgi) as the experimental and validation datasets, respectively^17^. We have corrected for batch effects using the sva_combat method. We used Perl 5 (v5.30.2) for all text processing. Compared to previous studies, we have constructed a prognostic model of Ewing's sarcoma using more sophisticated and accurate bioinformatics techniques for predicting prognosis. The turning language R was used for processing and graphing the data. All downloaded and completed data were normalized and log2 processed.

### Extraction of m6A-related genes, differential expression analysis, and correlation analysis

To analyze the differential expression of these genes, we used the “limma” packages to perform differential gene expression analysis. We compared the gene expression of Ewing's sarcoma samples with controls and set P < 0.05 as a significant differentially expressed gene. This was then visualized using the 'ggplot' package, the 'pheatmap' package and the 'ggpubr' package. The differential expression genes were visualized as heat maps and volcano plots. The differential expression of all m6A-related genes can be found in Table 1. Subsequently, the gene expression of m6A-related genes was extracted from 21,655 genes using the “limma” package. The “pheatmap” package was utilized to visualize them. The “ggpubr” and “reshape2” packages were utilized to obtain the m6A-related gene expression in ES and paraneoplastic tissues and visualized them as violin maps. Details of all m6A-related differentially expressed genes can be found in Table 1. Subsequently, the correlation of m6A-related differential genes was analyzed using the “corrplot” and “circlize” packages and visualized as correlation circle plots.

**Table 1:** Differential expression of m6A-related genes.

Gene	conMean	treatMean	logFC	*p*-value
METTL3	5.699018	7.14749	0.326723	1.50E-13
METTL14	4.58475	5.824376	0.34526	1.08E-15
WTAP	5.598477	6.282423	0.166287	1.05E-13
KIAA1429	4.444641	5.170975	0.21837	4.87E-14
RBM15	5.27808	6.091391	0.206758	1.08E-15
ZC3H13	6.066223	7.29778	0.266659	1.57E-11
YTHDC1	4.593895	5.222197	0.184939	1.08E-15
YTHDC2	4.840991	5.124283	0.082048	0.009839
YTHDF1	7.71216	8.424596	0.127472	1.22E-09
YTHDF2	7.580526	8.96268	0.241632	1.08E-15
HNRNPC	6.54433	7.751271	0.244187	1.08E-15
FTO	7.779931	7.944228	0.03015	0.068138
ALKBH5	7.533025	6.182872	-0.28495	2.16E-15

Table shows the differential expression of all m6A-related genes in Ewing's sarcoma.

### Construction of a prognostic model for Ewing’s sarcoma

Several techniques were used to analyze the relationship between m6A differentially expressed genes and ES prognosis to determine those closely associated with prognosis. First, the least absolute shrinkage and selection operator (LASSO) regression analysis^18,19^ method was used for maximum model refinement. This advanced, streamlined modeling approach compresses the coefficients of insignificant variables to zero; thus, fulfilling the variable selection effect. Subsequently, a multivariate COX regression analysis of survival status and time was used along with gene expression to obtain a more accurate prognostic model. After these two precise screening steps, we constructed a logistic regression model for m6A-related Ewing's sarcoma. Finally, a risk score was assigned to each Ewing's sarcoma case and, based on its median value, cases greater than or equal to the median value were classified as high risk and those less than the median value as low risk.

### The three-dimensional (3D)-principal component analysis, gene expression analysis, and risk scoring

**Figure 1:**
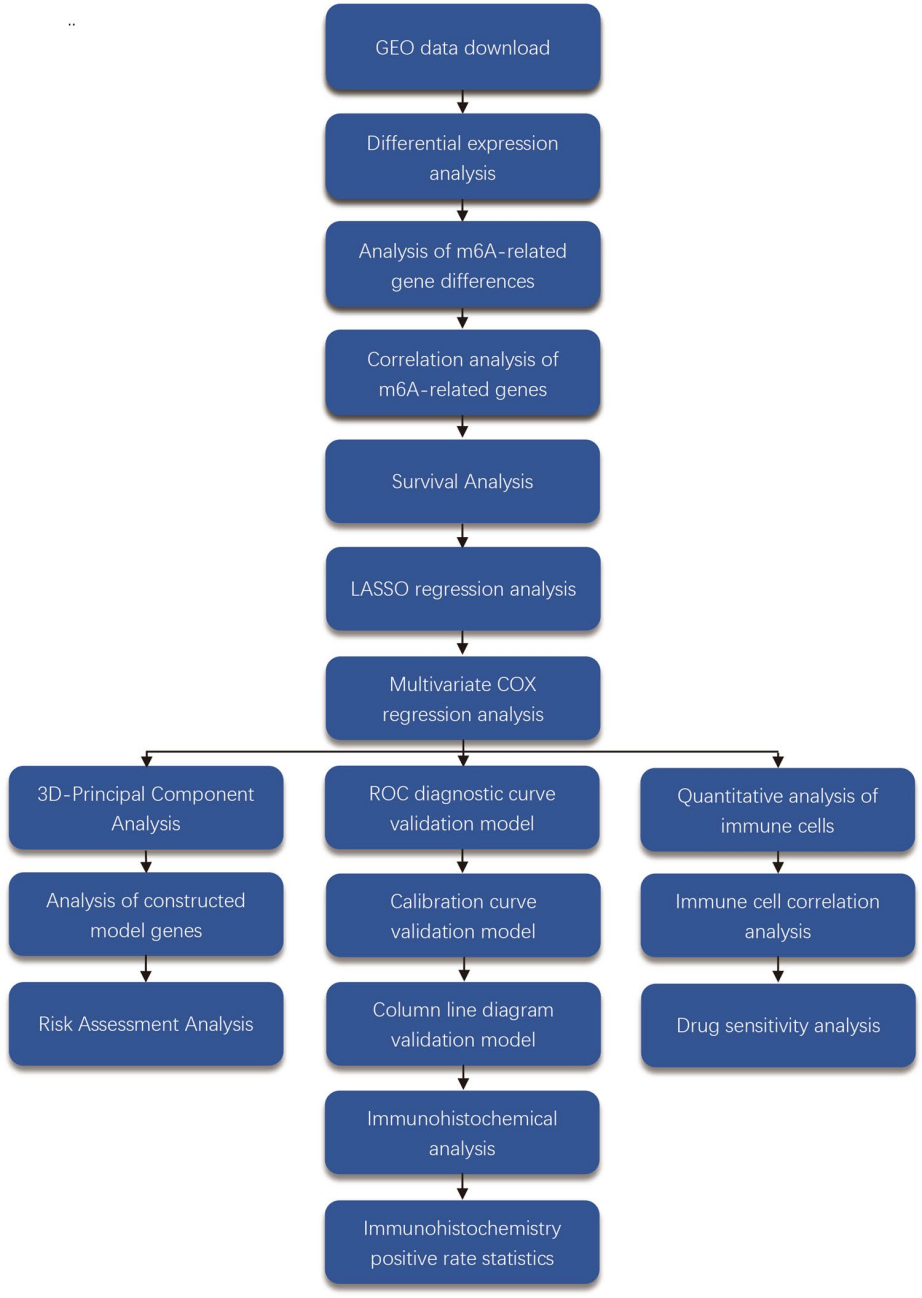
Workflow diagram. From the diagram, we can see the flow of our work clearly and briefly.

3D-Principal Component Analysis is a commonly used tool for data analysis. For a set of data that may be linearly correlated between different dimensions, PCA is able to transform this set of data into data that is linearly uncorrelated between the dimensions by means of an orthogonal transformation^20–22^. After an ES prognostic model was constructed, a 3D-principal component analysis was performed to analyze whether the two could be distinguished based on high and low-risk groupings. The “scatterplot3d” package was utilized to analyze and visualize it. Subsequently, based on the constructed prognostic model, the “ggpubr” package was used to analyze gene expression in-depth. All cases were ranked serially from low to high risk, and the corresponding survival status for each patient was plotted. Moreover, the expression of the genes used to construct the prognostic model in the high-and low-risk groups was analyzed (Fig. 1A–C).

### Survival analysis

Survival analysis was conducted for all cases to analyze the role of m6A-related genes in of sarcomas prognosis. These were analyzed using two different methods. First, based on the high and low expression values of each m6A-related gene, all patients were divided into high and low expression groups, and Kaplan–Meier survival curves were plotted. Subsequently, based on the constructed prognostic model, these curves were analyzed for all cases by dividing them into high-and low-risk groups. To verify the accuracy of the constructed prognosis, the same approach was utilized for GSE63157 to construct a prognostic model, yielding a Kaplan–Meier survival curve for the validated gene set.

### Examination of the prognostic model of Ewing’s sarcoma

The accuracy of our constructed prognostic model of ES was tested using the receiver operating characteristic (ROC) diagnostic and calibration curves as well as the column line graph method for testing the model. First, the ROC diagnostic curves were plotted for the model using the “survival”, “survminer”, and “timeROC” packages. Second, the ROC diagnostic curves of clinical information were prepared using these three R package pairs. Finally, the “rms” package was utilized for the calibration curves and column plots.

### Immune cell composition analysis and correlation analysis

In this study, all cases of ES were quantified to assess the relationship between the samples and immune cell composition using CIBERSORTs software^23–25^. The immune cells of each sample of ES were set to 100%, and the composition of a total of 22 immune cells was analyzed and visualized as a composition map. Subsequently, the relationship between these immune cells was assessed, and a correlation analysis was performed using the “corrplot” package. In contrast, we performed an in-depth analysis of m6A-related gene expression and immune cell content in ES to analyze the role of immune cells at multiple levels and perspectives.

### Drug sensitivity analysis

CellMiner software is based on 60 cancer cells listed by the National Cancer Institute's Center for Cancer Research (NCI). The NCI-60 cell line is currently the most widely used cancer cell sample population for anti-cancer drug testing^26,27^. It allows researchers to query data on the 22,379 genes identified in the NCI-60 cell line, as well as the 20,503 compounds analyzed (including 102 drugs approved by the US Food and Drug Administration). Here, a drug sensitivity analysis was conducted to investigate the relationship between two m6A genes and drug sensitivity for better guidance of clinical drug use. All data related to drug sensitivity was downloaded from the CellMiner database (version: 2021.1, database: 2.6). Subsequently, the programming language R (× 64, version 4.0.2) was used to assess the relationship between m6A-related gene expression information and drug sensitivity using the “impute,” “limma,” “ggplot2,” and “ggpubr” packages.

### Immunohistochemical analysis

The accuracy of the bioinformatics analysis was determined by performing immunohistochemical analysis on the two genes that were used for model construction. The pathological sections for immunohistochemistry were excised specimens obtained for pathological testing during surgery at the First Clinical Affiliated Hospital of Guangxi Medical University. The study followed the Declaration of Helsinki and was approved by the ethics department of the First Clinical Affiliated Hospital of Guangxi Medical University, and immunohistochemical staining analysis was performed on anonymous tissue specimens. Therefore, this study needs for the patient's written informed consent is waived. The immunohistochemical analysis was performed for a total of 12 pathological sections for each gene in three pairs (three each of cancer and para cancer tissue sections). Antibodies for immunohistochemical specific staining were purchased from Proteintech (https://www.ptgcn.com/) (under item numbers 26158–1-AP and 24744–1-AP). All sections were dewaxed and hydrated by cutting 3 µm thick paraffin sections, baking them overnight at 64 °C, dewaxing in water, and immersing in distilled water. Subsequently, antigen repair was performed, endogenous peroxidase was blocked, water was shaken off the sections, and sections were circled at 3 mm from the tissue using an immunohistochemical grease pencil. The peroxidase blocker was added dropwise to the sections for 10 min at room temperature and washed thrice for 3 min each in phosphate-buffered saline and 95%, 100%, and 100% alcohols. The slices were then sealed using neutral environmental protection resin. All stained pathological tissue sections were placed under an inverted microscope for observation and image acquisition. Subsequently, a statistical analysis of the positive regions of specific staining was performed for each gene. The positive rates for all immunohistochemically stained images were calculated using Image J software. Moreover, the data were imported into IBM SPSS Statistics 25, where they were analyzed using a t-test of two-paired sample means. Finally, the results of the statistics were visualized using GraphPad Prism 8.

### Quantitative reverse transcription PCR (qRT-PCR)

The normal human 293 T cells used in this study were purchased from Shenzhen Aowei Biotechnology, human Ewing's sarcoma cells A673 were purchased from SHANGHAI WHELAB BIOSCIENCE LIMITED and human Ewing's sarcoma cells RD-ES were purchased from Zhejiang Meisen Cell Technology Ltd., Co. 293 T cells and A673 cells were cultured in Dulbecco’s modified Eagle’s medium (DMEM) containing 10% FBS, 100 U/mL penicillin, and 100 mg streptomycin at 37 °C and 5% CO2 atmosphere. A673 cells were cultured in RPMI medium 1640 containing 10% FBS, 100 U/mL penicillin, and 100 mg streptomycin at 37 °C and 5% CO2 atmosphere. RNA extraction using Hipure Total RNA Mini kit (Magen, China) followed by quantitative real-time PCR (qRT-PCR) was used to purify the total intracellular RNA from the induced samples. Subsequently, 1000 ng of the extracted RNA was reverse transcribed into cDNA using a cDNA synthesis kit (Takara, China). qRT-PCR was used to detect the gene expression using the LightCycler 480 Sequence Detention System (Roche, Germany) and PCR Green Master Mix (Roche, Germany). The activation cycle of the polymerase included 10 min at 95 °C and15 s at 95 °C, and 45 cycles such cycles were performed. Glyceraldehyde 3-phosphate dehydrogenase (GADPH, Abcam, USA) was used as the internal control, and the data analysis was performed using the 2-ΔΔCT method. The analysis for each gene was performed in triplicate. The primer sequences of the target genes are presented in Table 2.

**Table 2:** Primer sequences for GAPDH and METTL14.

Primer	Sequence (5'–3')
METTL14-F	GGCTATGACTCCTAATCACGCTTCC
METTL14-R	ATCCAAGTTCAAGTCCACACCACAG
GAPDH -F	CCACTCCTCCACCTTTGAC
GAPDH -R	ACCCTGTTGCTGTAGCCA

Table shows the primer sequences for GAPDH and METTL14 during the primer design phase.

### Financial and competing interests disclosure

There were no conflicts of interest between all co-authors of this study. The authors have no relevant affiliations or financial involvement with any organization or entity with a financial interest in or financial conflict with the subject matter or materials discussed in the manuscript. This includes employment, consultancies, honoraria, stock ownership or options, expert testimony, grants or patents received or pending, or royalties. No writing assistance was utilized in the production of this manuscript.

### Ethical disclosure

This study was approved by the Ethics Review Committee of the First Clinical Affiliated Hospital of Guangxi Medical University and was in accordance with the Declaration of Helsinki of the World Medical Associatio.

### Data sharing statement

The datasets supporting the conclusions of this article are available in the GEO database (GSE21257:https://www.ncbi.nlm.nih.gov/geo/query/acc.cgi?acc=GSE21257, and GSE63157: https://www.ncbi.nlm.nih.gov/geo/query/acc.cgi?acc=GSE63157).

## Results

### Data download and preliminary processing

**Figure 2:**
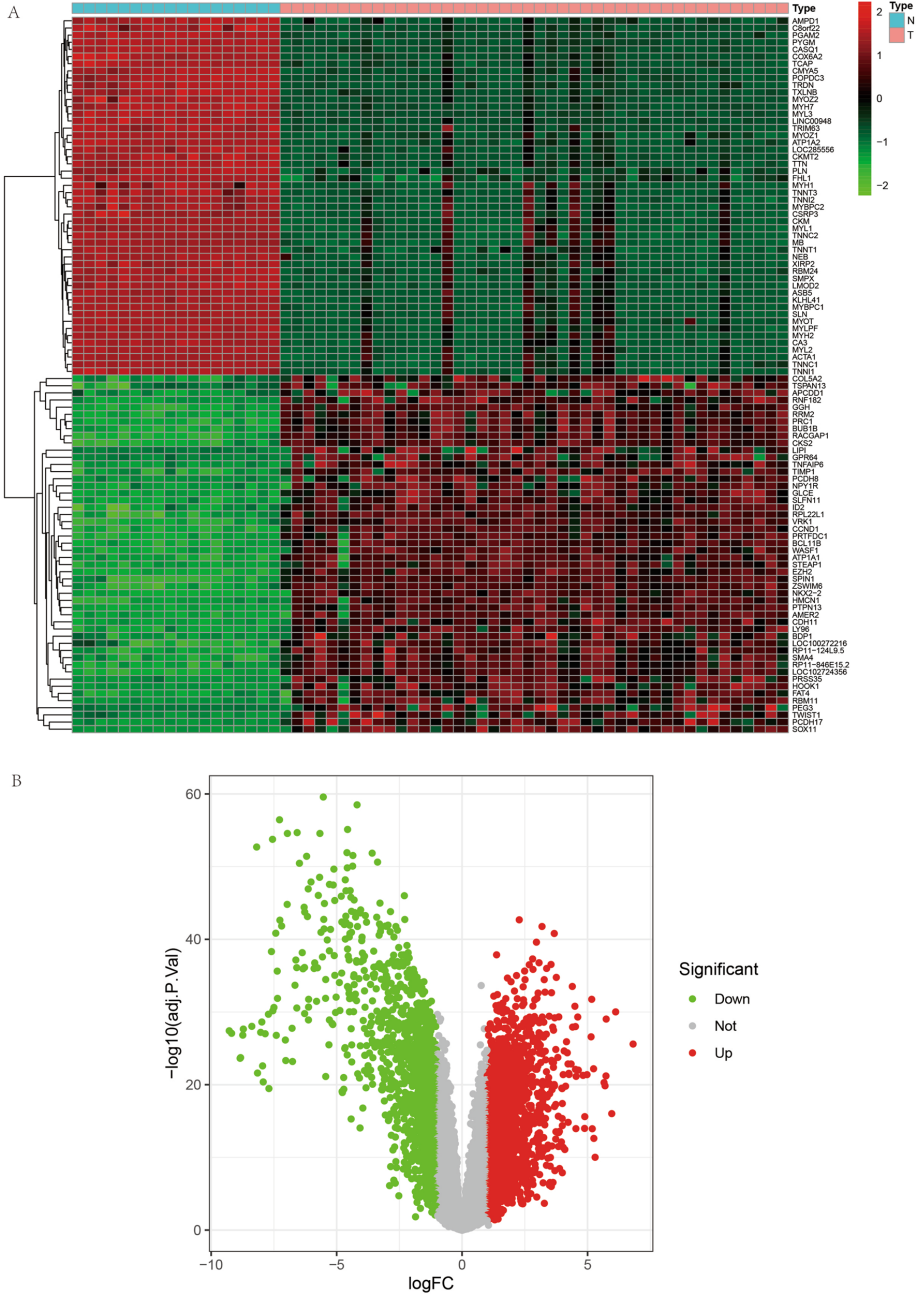
Heat map and volcano plots of differentially expressed genes. (**A**) shows a heat map of the differentially expressed genes. (**B**) shows the volcano map of differentially expressed genes.

We downloaded two datasets from the GEO database, GSE17674 as the experimental set for constructing the prognostic model for ES and GSE63157 as the validation set for the prognostic model. Among them, GSE17674 comprised 44 ES and 18 normal samples. A total of 85 ES samples were included in GSE63157 as the validation set. We analyzed a total of 147 samples from 129 ES and 18 normal control samples. All gene expression data were normalized using R to remove inter-batch differential processing. The flow chart is illustrated in Fig. 2.

### Differential expression analysis, extraction of m6A-related genes, and correlation analysis

**Figure 3:**
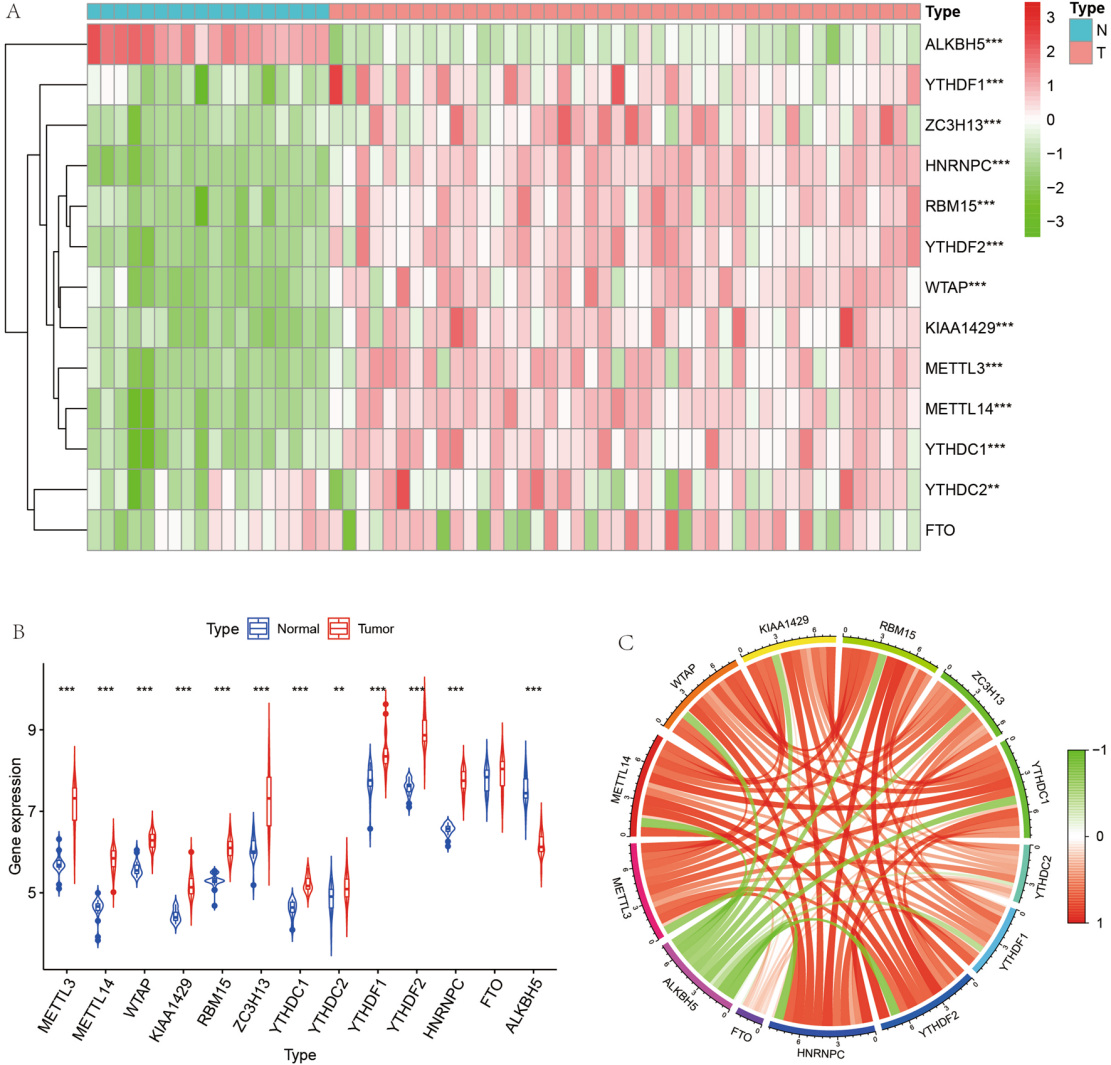
Heat map, violin map and correlation circle map of m6A-related genes. (**A**) shows a heat map of differential expression of m6A-related genes, with red indicating highly expressed genes and green indicating lowly expressed genes. (**B**) represents the expression of m6A-related genes in paraneoplastic tissues and in Ewing's sarcoma. Figure **C** represents the correlation plot between m6A-related genes, with the linkage between quantitative genes in red for synergistic high expression and in green for synergistic low expression.

**Figure 4:**
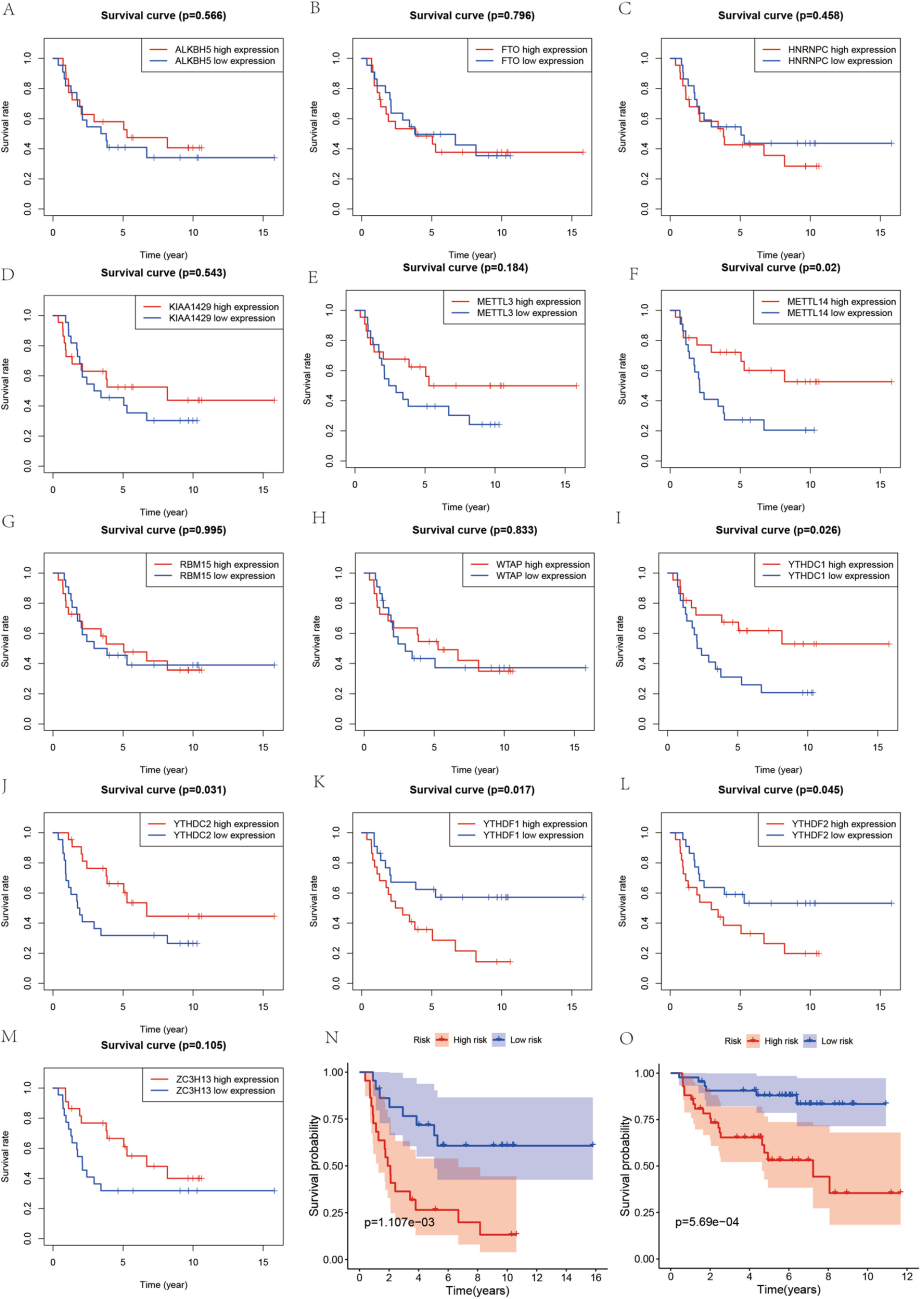
Survival analysis. (**A**–**L**) and (**M**) represent the results of survival analysis based on high and low expression of genes in Ewing's sarcoma. (**N**) represents the results of the constructed prognostic model. (**O**) represents the prognostic model validation of the validation set.

We first performed differential expression analysis on all genes and obtained a total of 3947 differentially expressed genes according to the set cut-off values. Heat map and volcano plots are shown in Fig. 3A, B. Subsequently, we extracted these genes from all gene expression matrices for subsequent analysis to analyze the role of m6A genes in ES. We performed differential expression analysis of these m6A-related genes (Fig. 4A) and observed that the differences in 12 of these 13 genes were statistically significant (P < 0.05). We analyzed the expression of m6A-related memory in tumor and normal samples and found that the expression of all 12 genes was higher in tumor samples than in normal samples except for ALKBH5 (Fig. 4B). We found that there was a correlation between both these m6A-related genes, with some showing synergistic high expression (Fig. 4C) and some showing low synergistic expression. We have placed details of the m6A-related differentially expressed genes in Table 1.

### Construction of a prognostic model for Ewing’s sarcoma

**Figure 5:**
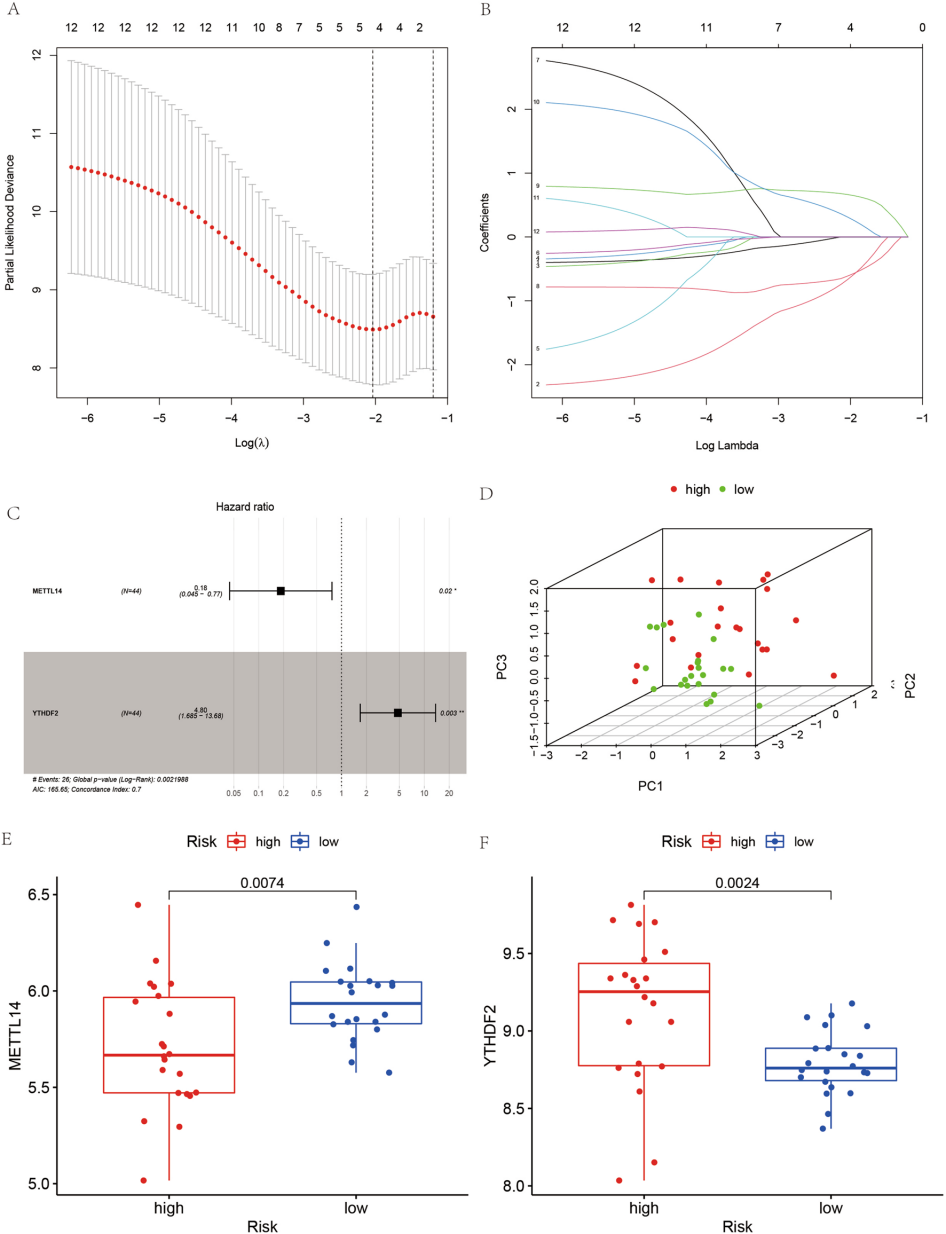
Diagram of the construction process of the prognostic model. (**A**) and (**B**) represent the results of the LASSO regression analysis. (**C**) represents a plot of the results of the multivariate COX regression analysis. (**D**) shows the 3D-principal component analysis plot, with red points indicating high-risk cases and green points indicating low-risk cases. (**E**) and (**F**) indicate the expression of METTL14 and YTHDF2 in the high-risk group and in the low-risk group, respectively.

We first performed LASSO regression analysis on these 13 m6A-related genes to maximize the number of genes in the prognostic model and obtain the most accurate and streamlined prognostic model. As observed from the results of the LASSO regression analysis (Fig. 5A and B), the most streamlined results can be achieved when the number of genes is four. From the multivariate COX regression analysis (Fig. 5C), we found that after analysis, only two genes, METTL14 and YTHDF2, were closely associated with the presence of survival. Details of the results of the multivariate COX regression analysis can be found in Table 3. We constructed a prognostic model for ES based on these two genes, and each case was rated for risk. The cases with risk scores greater than or equal to the mean were classified into the high-risk group and those less than the mean into the low-risk group.

**Table 3:** Results of the multivariate COX regression analysis.

ID	Coef	HR	HR.95L	HR.95H	*p* value
METTL14	−1.6877	0.184944	0.044527	0.768168	0.02018
YTHDF2	1.569026	4.801968	1.685304	13.68234	0.003314

Table shows the detailed results of the multivariate COX regression analysis.

### 3D-principal component analysis, gene expression analysis, and risk scoring

From the constructed 3D-principal component analysis plot, we observed (Fig. 5D) that the cases in the high-risk group were distributed in the positive axis direction of the PC3 axis, while most of the cases in the low-risk group were distributed in the negative axis direction of the PC3 axis. Moreover, we found that the gene expression value of METTL14 was lower in the high-risk group than that in the low-risk group (Fig. 5E), with a statistically significant difference. The gene expression value of YTHDF2 was higher in the high-risk group than that in the low-risk group (Fig. 5F), with a statistically significant difference. Each case was ranked consecutively based on the risk score from the lowest to the highest to obtain the risk graph (Fig. 1A–C).

### Survival analysis

**Figure 6:**
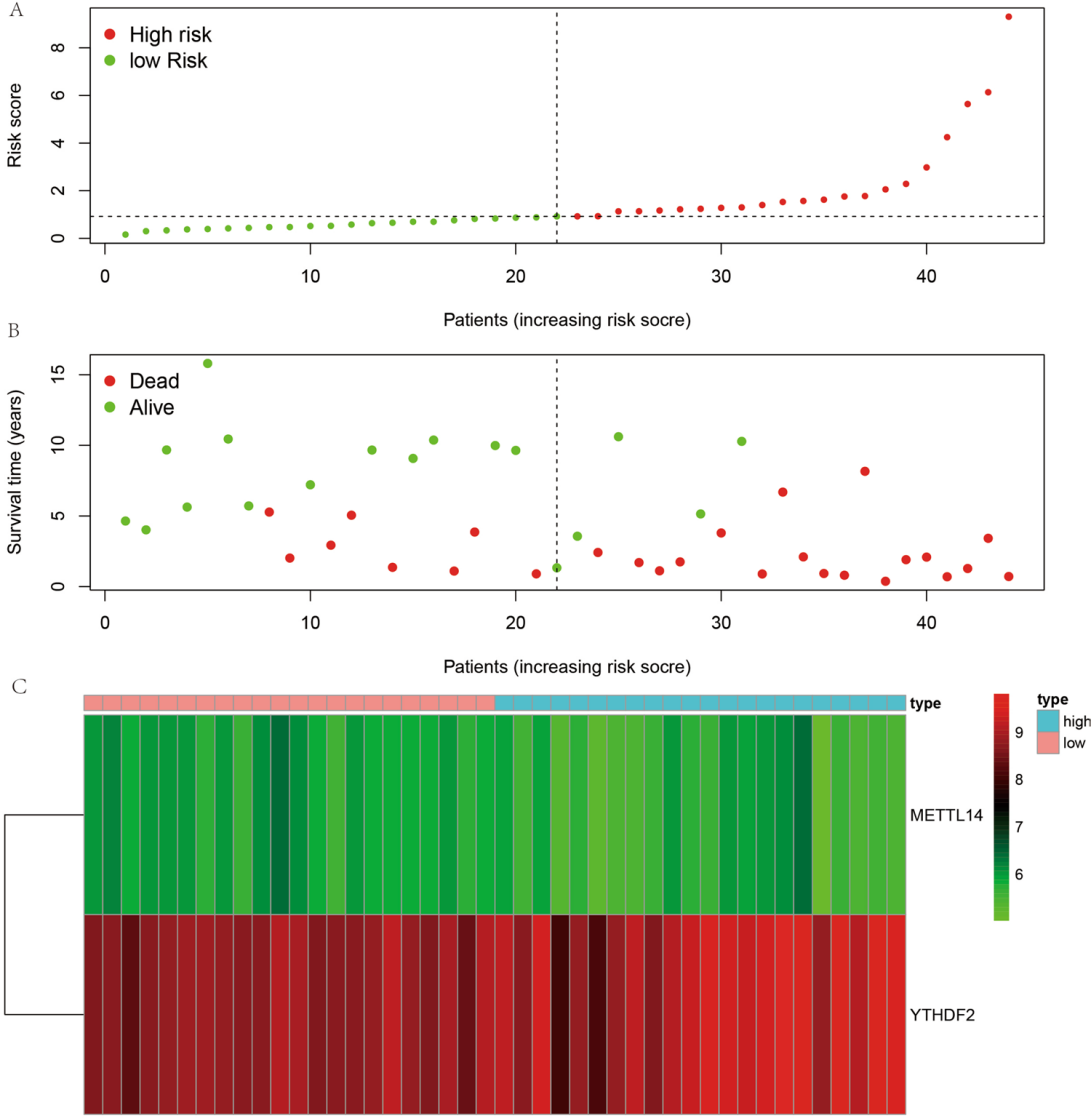
Risk graph for Ewing's sarcoma cases. (**A**) represents the risk score chart based on the risk of the cases in order from ground to high. (**B**) represents the graph of survival and death of the cases. (**C**) represents the expression of METTL14 and YTHDF2 in high and low risk groups.

We first divided all cases into the high and low expression groups of this gene for Kaplan–Meier survival curves based on the mean expression of each m6A gene to utilize the survival data of ES completely. We observed that except for METTL14, YTHDC1, and YTHDF2 (Fig. 6A–M), the differences between the other ten genes in these two groups were not statistically significant (*P* > 0.05). Moreover, the results of our constructed prognostic model for m6A-associated ES showed that cases in the high-risk group possessed a worse survival rate than those in the low-risk group (Fig. 6N), with a statistically significant difference (*P* < 0.05). Also, we constructed a prognostic model using the same method for GSE63157, yielding similar results (Fig. 6O), with cases in the high-risk group having worse survival than those in the low-risk group (*P* < 0.05).

### Examination of the prognostic model of Ewing’s sarcoma

**Figure 7:**
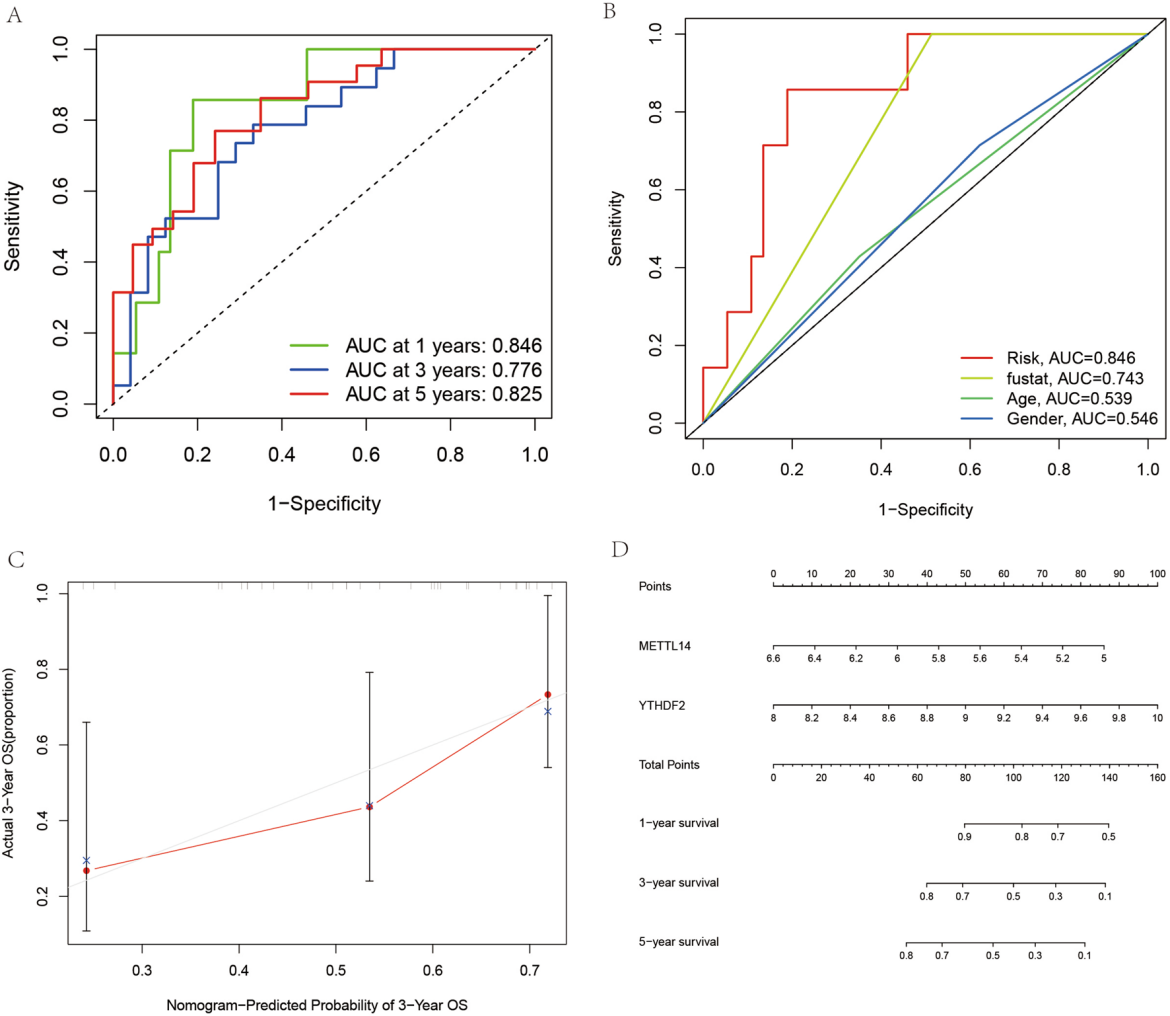
A test plot of the prognostic model of Ewing's sarcoma. (**A**) represents the accuracy of predicting the probability of survival at 1,3 and 5 years. (**B**) indicates the accuracy of the diagnostic clinical information. (**C**) represents the calibration curve for predicting patient survival. (**D**) represents the column line plot for predicting the probability of patient survival.

The ROC diagnostic curve indicated (Fig. 7A) that the constructed prognostic model predicts the 1-year, 3-year, and 5-year survival rates with an accuracy above 50%. Also, as depicted in Fig. 7B, the area under the curve of the ROC curve for the diagnosis of risk, survival status, age, and sex with clinical information is greater than 0.5. The calibration plot depicts (Fig. 7C) that the starting point and focus of the predicted survival largely overlap, which further illustrates the accuracy of our constructed prognostic model. In contrast, we constructed a line graph (Fig. 7D) and were able to predict the survival rate of patients more accurately using the line graph.

### Immune cell composition analysis and correlation analysis

**Figure 8:**
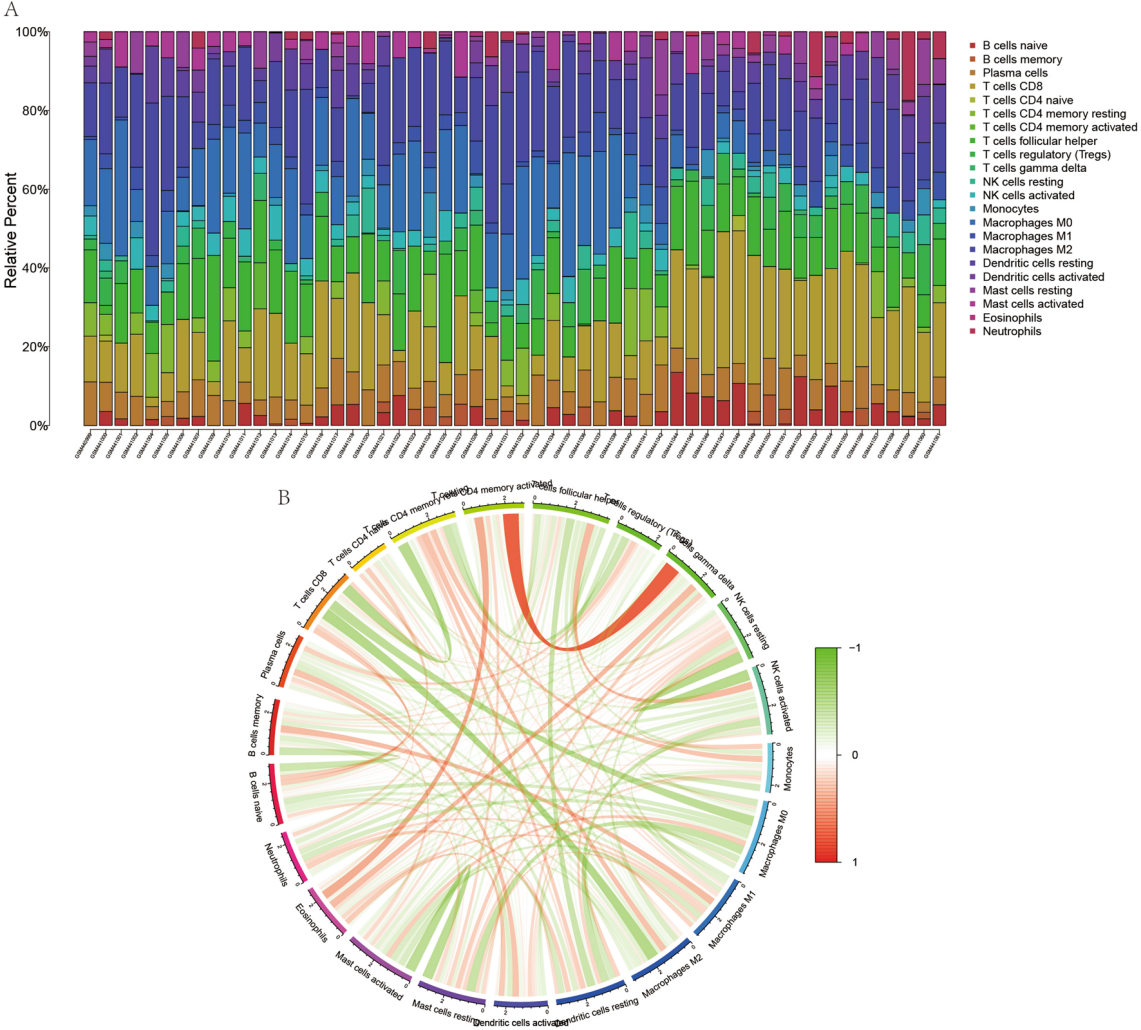
Immune cell composition and correlation analysis. (**A**) represents the analysis of immune cell composition for all cases, with each color indicating one immune cell. (**B**) represents the correlation analysis between immune cells, with red connecting lines indicating synergistic high expression and green links indicating synergistic low expression.

**Figure 9:**
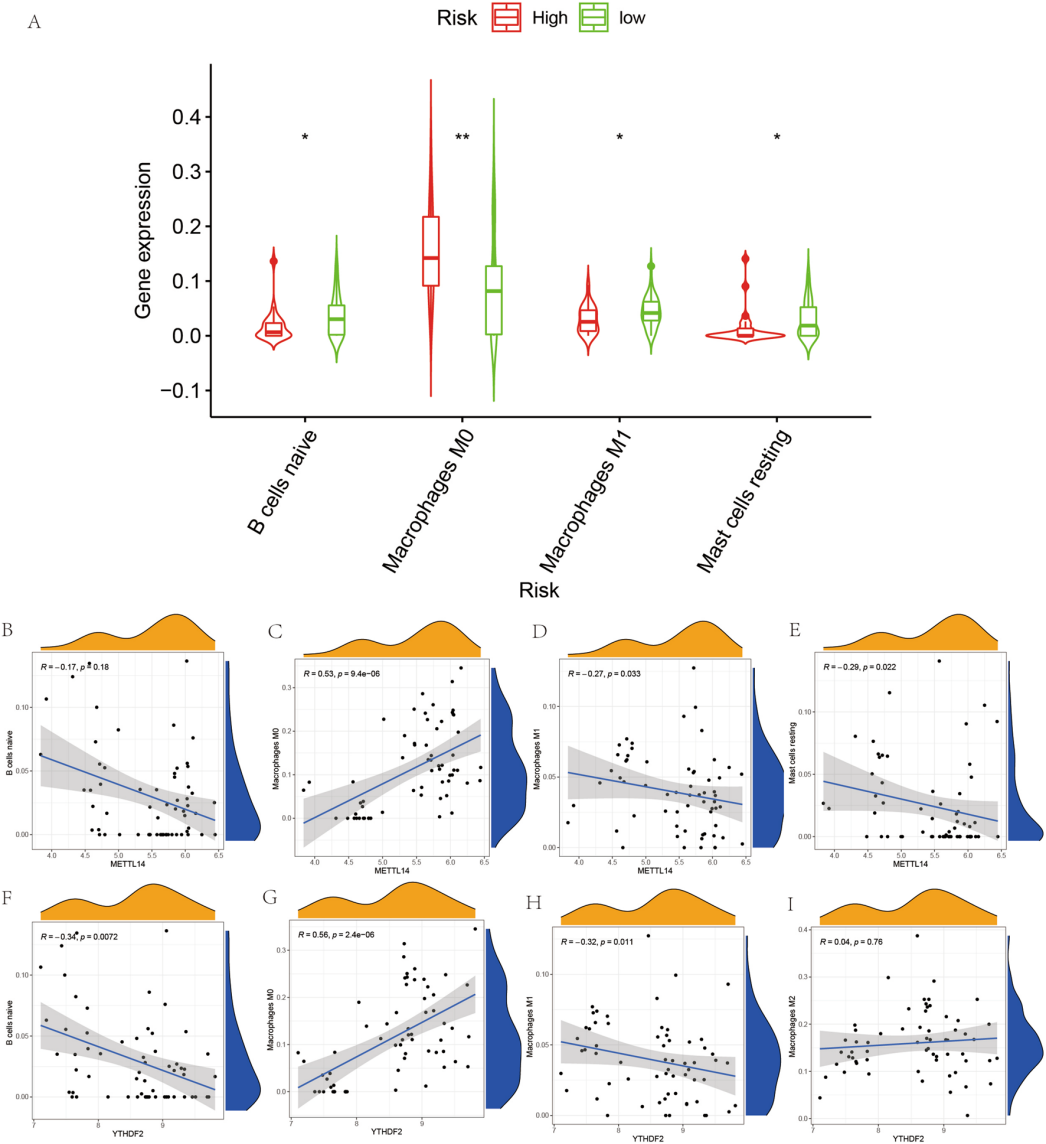
Expression of immune cells in Ewing's sarcoma and paraneoplastic tissues. Figure A shows the expression of four immune cells in Ewing's sarcoma and paraneoplastic tissues, "*" indicates *P* < 0.05, "*" indicates *P* < 0.01. (**B**–**E**) represent the correlation analysis obtained between METTL14 and this B cells naive, Macrophages M0, Macrophages M1 and Mast cells resting. (**F**–**H**) and (**G**) represent the correlation analysis obtained between YTHDF2 and this B cells naive, Macrophages M0, Macrophages M1 and Mast cells resting.

We used CIBERSORT software to quantify the composition of immune cells in both ES and normal cases. This is illustrated in Fig. 8A, with each sample comprising 22 types of immune cells. Moreover, there is a correlation between the expression of these 22 immune cells (Fig. 8B), with the red line synergizing high expression and green synergizing low expression. We performed a differential analysis of immune cell components in all cases (Fig. 9A) and found significant differences in the expression of naïve B cells, macrophages M0, macrophages M1, and resting mast cells in ES and normal controls (*p* < 0.05). Also, we analyzed the relationship between METTL14 and YTHDF2 with these immune cells (Fig. 9B–E) and found that METTL14 showed a negative correlation with naïve B cells, macrophages M1, and resting mast cells, and a positive correlation with macrophages M0 (*P* < 0.05). In contrast (Fig. 9F–I), YTHDF2 showed a negative correlation with naïve B cells and macrophages M1 and a positive correlation with macrophages M0 and resting mast cells (*P* < 0.05).

### Drug sensitivity analysis

**Figure 10:**
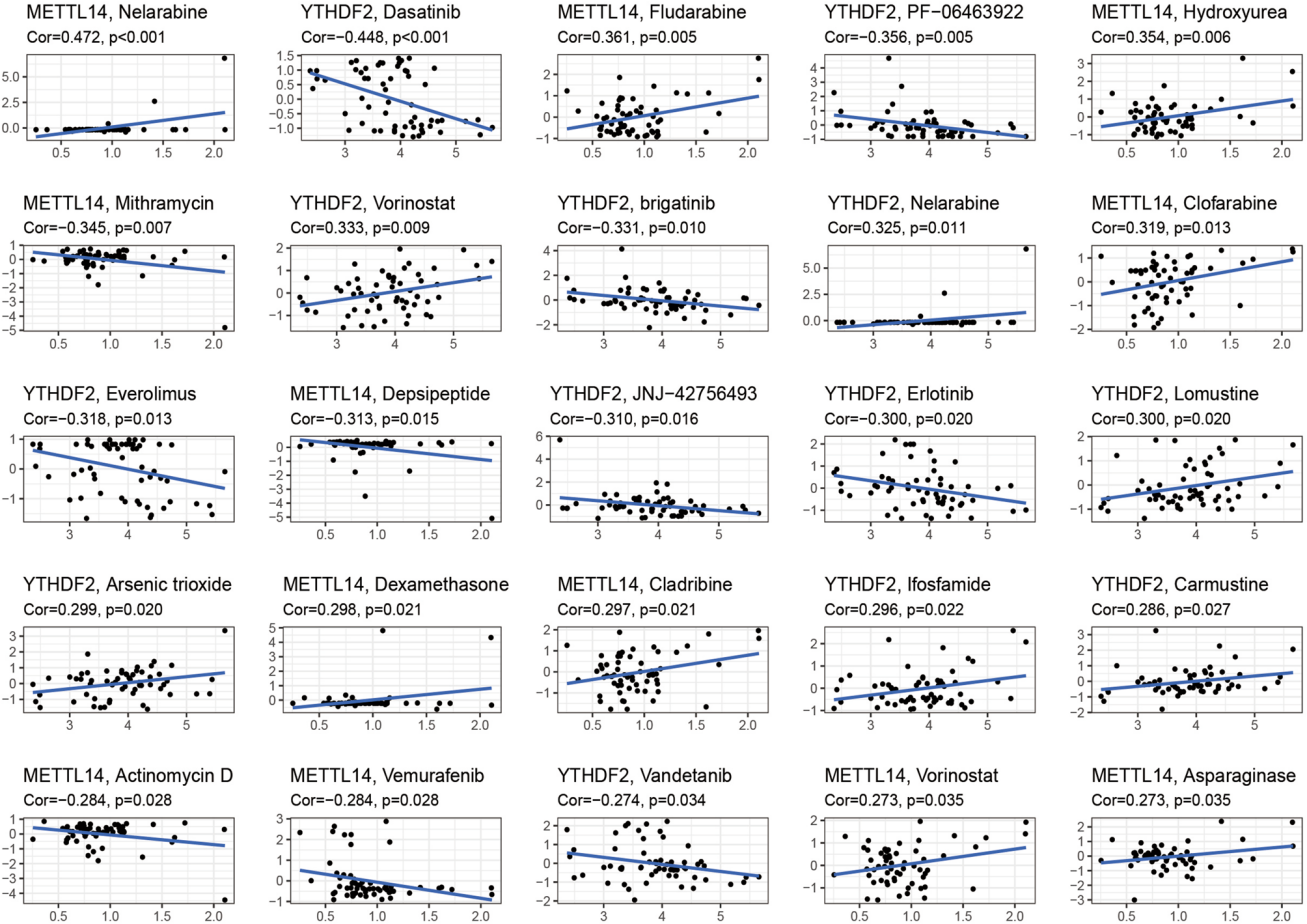
Drug sensitivity analysis. The figure shows the relationship between METTL14 and YTHDF2 each with different drug sensitivities. If Cor > 0, the high expression of this gene showed a positive correlation with drug sensitivity. If Cor < 0, the high expression of this gene showed a negative correlation with drug sensitivity.

To better explore the relationship between Ewing's sarcoma and drug sensitivity, a drug sensitivity analysis was performed. Using drug sensitivity analysis, we found that METTL14 and YTHDF2 were quite closely related to the drug sensitivity of multiple drugs (Fig. 10). Among these, METTL14 showed a positive correlation with the drug sensitivity of several drugs, including Nelarabine, Hydroxyurea, and Clofarabine, and a negative correlation with the Mithramycin, Depsipeptide, and Actinomycin. In contrast, YTHDF2 was closely associated with the sensitivity of several drugs such as Dasatinib, Vorinostat, and Brigatinib. A positive correlation indicates that the sensitivity of the corresponding drug increases with the increase in the gene expression value. A negative correlation indicates that the sensitivity of the corresponding drug is weakened with the increase in the gene expression value.

### Immunohistochemical analysis

**Figure 11:**
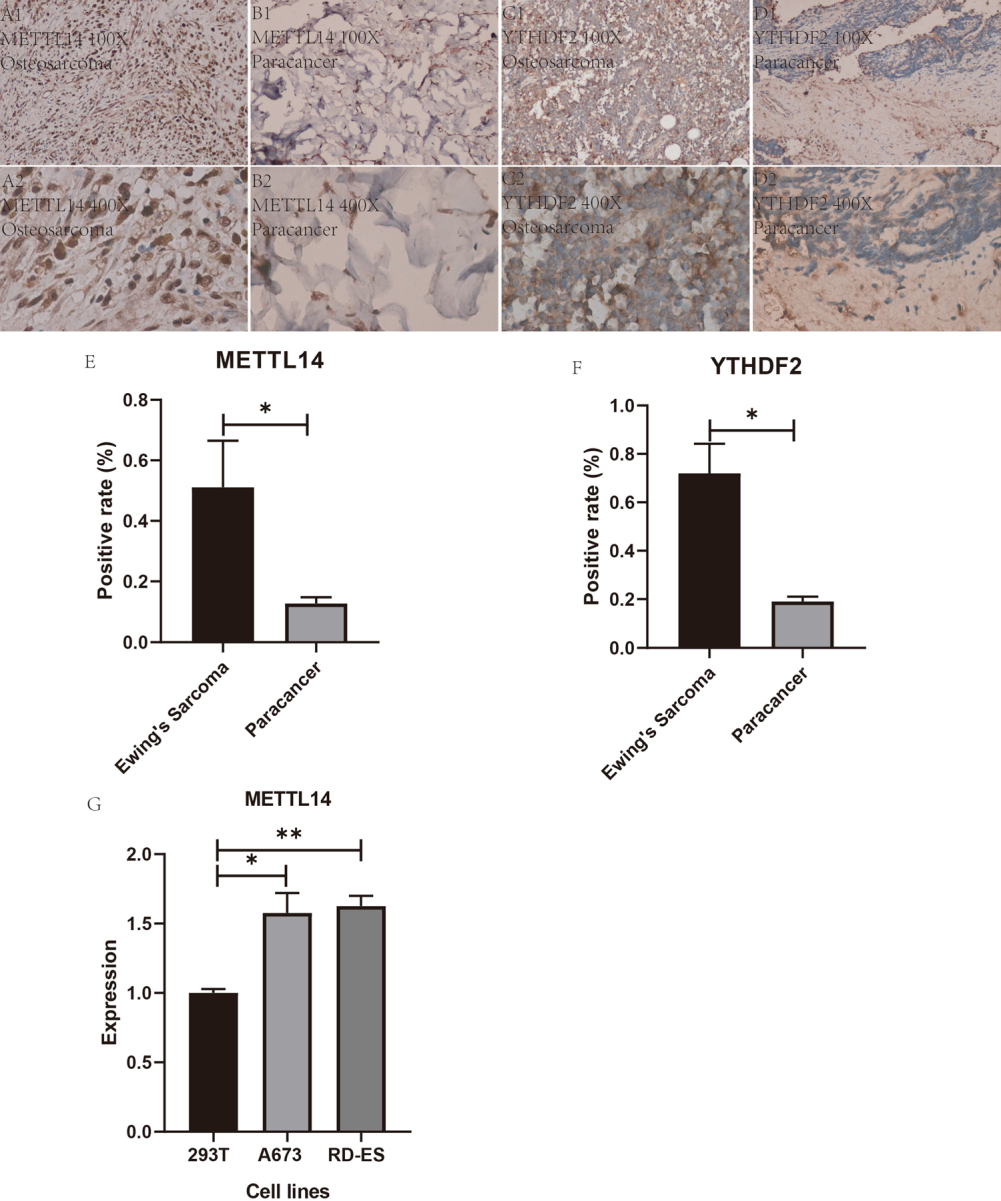
Immunohistochemistry, positivity statistics and qRT-PCR results. (**A1**, **A2**, **B1**, **B2**) represent immunohistochemical specific staining maps of METTL14 in Ewing's sarcoma tissue and in paraneoplastic tissue at 100× magnification and 400× magnification. (**C1**, **C2**, **D1**, **D2**) represent immunohistochemical specific staining maps of YTHDF2 in Ewing's sarcoma tissue and in paraneoplastic tissue at 100× magnification and 400× magnification. (**E**) indicates the statistical plot of the positive rate of immunohistochemistry for METTL14. Figure F represents the statistical graph of the positive rate of immunohistochemistry for YHHDF2, "*" indicates *P* < 0.05. (**G**) shows the expression of METTL14 in two Ewing's sarcoma cell lines and one control cell line, which can be found to be significantly different in ES and control."*" indicates *P* < 0.05. "**" indicates *P* < 0.01.

We performed laboratory analysis of six (three cases of ES and three cases of paraneoplastic tissue) of each gene for a total of 12 immunohistochemical specific stains. After laboratory manipulation, we obtained all the specific stained sections, which we placed in the same inverted microscope for observation. We found that the specific expression of both METTL14 and YTHDF2 was higher in ES than in the paraneoplastic tissues (Fig. 11A1–D2). We were able to find from the pictures of the specific staining by immunohistochemistry that the specific expression of both METTL14 and YTHDF2 in Ewing's sarcoma was higher than their respective expression in the control group. This is consistent with our previous analysis and further enhances the credibility of the prognostic model we have constructed. We again performed statistical analysis of the slices using Image J software for positive rate statistics, using the IBM SPSS Statistics 25 t-test for two-paired sample means. The positive rates of both METTL14 and YTHDF2 (Fig. 11E, F) were observed to be higher in ES than in the paraneoplastic tissues (*P* < 0.05).

### Quantitative reverse transcription PCR (qRT-PCR)

We performed in vitro qRT-PCR cellular assays for cellular level validation of the METTL14 gene in order to verify the reliability of the bioinformatics analysis. From Fig. 11G, we can learn that the expression of METTL14 gene in the control cell line 293 T was much lower than that in the human Ewing's sarcoma A673 cell line (*P* < 0.05); in addition, its expression in the control 293 T was also much lower than that in the human Ewing's sarcoma RD-ES (*P* < 0.01).

## Discussion

N6-RNA methylation modifications appear as a highly abundant dynamic regulation throughout eukaryotic transcription. With an increasing number of m6A-related genes observed to be dysregulated in tumors, m6A alters the course of cancer cell development^28^. The m6A regulators have been shown to promote the development of various types of cancer possibly, and a study by Anita et al. found that overexpression of YTHDF3 and YTHDF1 can lead to poor prognosis in patients with breast cancer^29^. Moreover, m6A-related genes were not only dysregulated in expression in breast cancer but also in gastric cancer. Wang et al. demonstrated that high expression of METTL3 promoted glycolysis and tumor angiogenesis in gastric cancer, suggesting METTL3 as a potential therapeutic target for human gastric cancer^30^. However, the role of m6A-related genes in ES is poorly reported, and the low prognosis and high metastatic nature of this malignant disease critically require investigators to explore the role of m6A-related genes in ES.

Methyltransferase Like 14 (METTL14) is a protein-coding gene that is most closely associated with periosteal chondrosarcoma and Miyoshi muscular dystrophy 3. The upregulation of METTL14 has been demonstrated to lead to the decrease of PERP level through m6A modification which promotes the metastasis and growth of pancreatic cancer; thus, METTL14 can be a potential therapeutic target for pancreatic cancer^31^. METTL14 has also been reported in leukemia; METTL14 exerts oncogenic effects by regulating its mRNAs (MYB and MYC) through m6A modifications^32^. Liu et al. found that approximately 70% of endometrial tumors exhibit reduced m6A methylation, which may be owing to mutations in METTL14 or reduced expression of METTL3 (another component of the methylation transferase complex), and that these changes lead to a possible increase in endometrial tumorigenicity^33^. Thus, the above study is consistent with our results. Our results show that the prognostic models constructed for m6A-related genes, including METTL14, have much lower survival rates in their high expression group than in the low-risk group. More importantly, we found that METTL14 showed a negative correlation with naïve B cells, macrophages M1, and resting mast cells and a positive correlation with macrophages M0 (*p* < 0.05), and these immune cells are closely related to cancer development^34–37^. This provides new evidence for immunotherapy of ES. Moreover, we performed a correlative drug sensitivity analysis of METTL14 and found that this gene showed a quite close correlation with the sensitivity of multiple drugs, providing new reference evidence for the treatment of ES.

YTH N6-Methyladenosine RNA Binding Protein 2 (YTHDF2), a member of the superfamily containing the YTH structural domain, is a typical structural domain of eukaryotes, whose structural domain is generally located in the middle of the protein sequence and may have a function similar to that of RNA nodules. It has been found that YTHDF2 can regulate m6A methylation of OCT4 mRNA to promote liver phenotype and cancer metastasis in cancer stem cells^38^. Dixit et al. found that the m6A-related gene YTHDF2 is aberrantly expressed in glioblastoma compared to normal neural stem cells and could be a therapeutic target for the treatment of MYC signaling in glioblastoma^39^. This is consistent with the results of our study. Our results showed that the prognostic model constructed by m6A-related genes, including YTHDF2, had a much lower survival rate in the high expression group than that in the low-risk group. Moreover, we found that this YTHDF2 is particularly closely related to naïve B cells, macrophages M0, macrophages M1, and resting mast cells, which are immune cells. This may provide a new reference for guiding the immunotherapy of ES. Also, we found a close correlation between the sensitivity of YTHDF2 and several drugs such as Dasatinib, Vorinostat, and Brigatinib, which provides new reference information for the drug treatment of ES.

Here, we downloaded two datasets from the GEO database, constructed m6A-related ES, prognostic models using LASSO and multivariate COX regression analysis, and analyzed the prognosis of ES by differential expression analysis, 3D-principal component analysis, gene expression analysis, survival analysis, risk assessment of ES cases, ROC diagnostic curve, calibration curve analysis, and column line graph prediction analysis. The roles of m6A-related genes METTL14 and TYHDF2 in ES were analyzed using precise and sophisticated bioinformatics techniques, such as immune cell composition, immune cell correlation, immune cell-gene correlation, and drug sensitivity analyses. METTL14 and TYHDF2 serve as biomarkers of Ewing's sarcoma, and we can use the expression of these two genes in Ewing's sarcoma to predict the survival risk and prognosis of Ewing's sarcoma patients, providing new and useful information to guide clinical diagnosis and treatment. Finally, we tested our results using two different levels of laboratory methods. First, we first performed immunochemical specific staining of Ewing's sarcoma specimens derived from human tissue, and our analysis showed that both genes were expressed more in Ewing's sarcoma than in the control group, and the differences were statistically significant (*P* < 0.05). Secondly, we performed cell cultures of Ewing's sarcoma and control cells and the results of the qRT-PCR experiments performed showed that METTL14 expression was higher in both Ewing's sarcoma cell lines than in the control cell lines, with statistically significant differences (*P* < 0.05). All experimental results support the results of our bioinformatics analysis. We therefore have reason to believe that METTL14 and YTHDF2 can serve as prognostic biomarkers for Ewing's sarcoma associated with m6A.

Similar to others, our study also had certain limitations. First, the sample size was inadequate. We used a total of 147 samples for analysis and validation, which was insufficient. Second, we did not perform an adequate analysis of the specific tumor staging of ES.

## Conclusion

A prognostic model constructed using the m6A-related genes METTL14 and TYHDF2, whose survival rates for cases in the high-risk group were much lower than those in the low-risk group, can be a potential prognostic biomarker for ES.
